# Photoluminescence Enhancement in CdSe/CdS Quantum Dot Colloidal Films Induced by Gold Nanoparticles (AuNPs)

**DOI:** 10.1021/acsomega.5c03718

**Published:** 2025-09-08

**Authors:** Maoz Maoz, Syed Abdul Basit Shah, Vanni Lughi

**Affiliations:** † Department of Engineering and Architecture, 19028 University of Trieste, Via Valerio 6/A, 34127 Trieste, Italy; ‡ Department of Information Engineering, Electrical Engineering, and Applied Mathematics (DIEM), 9315 University of Salerno, Invariante 12/B, Via Giovanni Paolo II, 132, 84084 Fisciano, Salerno, Italy

## Abstract

The optical properties of quantum dots (QDs) can be altered by employing surface plasmon metallic nanoparticles close to the QDs. In this study, we investigate the photoluminescence (PL) enhancement of CdSe/CdS core–shell QDs coupled with gold nanoparticles (AuNPs) due to the plasmonic effect. The effect of the morphology of AuNPs and the importance of the spacer layer were also analyzed. The AuNPs are deposited on a glass substrate by magnetron sputtering to achieve precise morphological control. The deposited nanoparticles have a uniform distribution and optimal particle size ranging between 10 and 12 nm. A poly­(methyl methacrylate) (PMMA) spacer layer was employed between QDs and AuNPs to control the separation and avoid quenching effects due to Förster resonance energy transfer (FRET). Maximum PL enhancement was observed for a spacer layer of 25 nm thickness due to the plasmonic effect. This coupled structure can potentially be used to enhance the PL of QDs acting as a downshifting layer, which can be used to improve the power conversion efficiency (PCE) and improve light trapping in solar cells.

## Graphical abstract

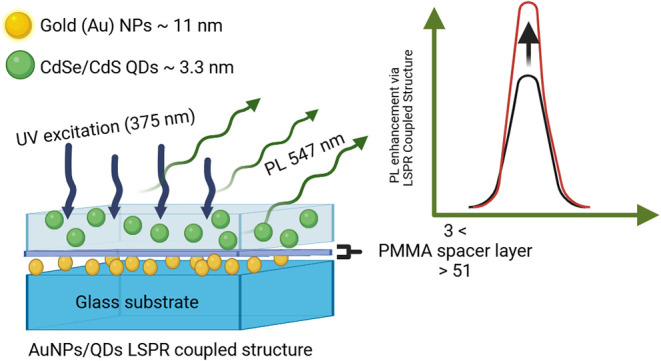

## Introduction

Enhancing the photoluminescence effect (PL) of quantum dots (QDs) has been a significant research attraction due to their promising applications in optoelectronics, more specifically in solar cells and LEDs, due to tunable emission bandgap, high quantum yield, and excellent photostability.^1^ Integration of QDs with plasmonic metallic nanoparticles (MNPs) has been a promising approach to enhance the optical properties of colloidal QD films.^2,3^ Among these, the CdSe/CdS core–shell structure has been extensively studied due to its excellent optical properties, high quantum yield, and tunable emission wavelengths across the visible range. This realizes its potential application as a UV downshifting layer in improving energy conversion efficiency in solar cells.^4,5^ Despite well-known attributes of these QDs, they experience few issues such as nonradiative recombination losses, PL quenching, and limited excitation efficiency, thereby restricting practical applications.^6^

Metallic nanoparticles (MNPs) are known to improve the photoluminescence of fluorophores such as quantum dots (QDs) owing to localized surface plasmon resonance (LSPR). The structure of coupled quantum dots (QDs) and metallic nanoparticles (MNPs) has emerged as a promising configuration to enhance the photoluminescence (PL) properties of quantum dots.^7^ This approach couples the unique properties of QDs with the localized surface plasmon resonance (LSPR) effects of AuNPs, resulting in increased excitation rates, improved quantum yields, and altered emission characteristics. The PL enhancement of QDs involves a complex mechanism and relies on different factors such as the plasmonic metal type, its morphology, the distance between the QDs and MNPs, the absorption and emission spectra of QD, and the excitation wavelength.^8,9^ The coupling mechanism can be categorized into three types. Local field enhancement near plasmonic nanoparticles raises the actual excitation strength felt by nearby quantum dots.^10^ This effect is strongest when the plasmon resonance matches the excitation wavelength and can give enhancement values of 2 to 10 times.^11^ Change of the local photonic state density by plasmonic nanostructures can speed up the radiative decay rate of quantum dots.^12^ This Purcell effect is strongest when the plasmon resonance overlaps with the QD emission wavelength.^13,14^ The interaction between quantum dots and gold nanoparticles can involve different energy transfer methods based on the separation distance and spectral overlap.^15^ Förster resonance energy transfer (FRET) is stronger at middle distances (2–10 nm) and shows a known 1/*r* ^6^ distance dependence.^16^ The space between quantum dots and plasmonic nanoparticles is likely the most important factor shaping the kind and strength of the interaction.^17^ At very short ranges (<5 nm), nonradiative energy transfer to the metal surface usually dominates, causing PL quenching.^18^ At middle distances (5–20 nm), a mix of enhancement and quenching can give overall PL improvement. At large distances (>30 nm), the plasmonic effects become small. Recent studies show that the best distance for PL enhancement is usually between 10–20 nm for gold nanoparticles. This range allows strong field enhancement while keeping energy loss to the metal low.^19^

CdSe/CdS core–shell QDs are especially attractive in the context that they have excellent quantum yield and photostability. The core CdSe absorbs UV photons, whereas the CdS shell acts as a passivation layer, thus reducing nonradiative recombination and in turn improving PL.^6^ The emission wavelength of these QDs lies in the visible region, which is well suited for silicon solar cells.^5^ The synthesis methods for CdSe/CdS QDs have grown from standard hot-injection techniques to more controlled approaches. Cirillo et al. showed a “flash” synthesis method that allows the quick growth of thick CdS shells up to 6.7 nm in just 3 min while keeping high photoluminescence quantum yields.^20^ Other low-temperature synthesis routes have also been made, with Zhang et al. reporting tetrapod CdSe/CdS QD synthesis at 120 °C using mixed amine ligands.^21^ The core–shell setup gives several benefits beyond surface passivation. The lattice mismatch between CdSe and CdS (3.9%) allows for proper shell growth while keeping the crystal structure stable.^1^

Magnetron sputtering gives several benefits for putting gold nanoparticles on quantum dot films. The method provides strong control over particle size, density, and spacing while staying compatible with various substrate materials.^22^ The sputtering settings, including power, pressure, time, and gas flow, can be finely adjusted to improve nanoparticle properties.^23^ Recent studies have shown the successful creation of gold nanoparticles using both DC and RF magnetron sputtering methods. The choice of the sputtering setup greatly affects the resulting nanoparticle shape and optical features. Low-power, short-time sputtering often makes small, well-spread nanoparticles, while higher power or longer times can cause larger particles or full film formation.^24,25^

Most of the research in this area is focused on employing colloidal chemical routes for AuNPs synthesis, which often exhibit limitations such as reduced reproducibility, less control over morphology, and scalability issues, all of which are critical for LSPR.^25–27^ In contrast, magnetron sputtering, a physical vapor deposition technique, gives remarkable control over the nanoparticle size distribution and uniformity, essential for enhancing plasmonic interactions. The use of magnetron sputtering provides a scalable and efficient method to fabricate these hybrid structures.^25,27,28^ The morphology of AuNPs is a critical parameter that affects PL enhancement. Larger particles show stronger plasmonic effects but can cause scattering losses. A trade-off must be made to achieve the optimal size and density of metal nanoparticles for enhancing the PL effect.^26^ Another important factor that plays a vital role in enhancing PL is the distance between the QDs and AuNPs. To allow for precise separation between the two components, a thin spacer layer (PMMA, SiO_2_) is often introduced, which ensures optimal energy transfer while avoiding quenching effects. The thickness of the spacer layer can be adjusted to balance the FRET and plasmonic effects.^17,26,29^

Despite the demonstration of PL enhancement using AuNPs being known, our study employs magnetron sputtering for AuNPs, which provides higher reproducibility and morphological control, addressing the limitation of colloidal synthesis. Furthermore, the use of AuNPs deposited via magnetron sputtering for enhancing the PL of the CdSe/CdS core–shell structures has not been previously investigated. This requires further investigation, particularly regarding the optimized AuNP morphology and spacer layer thickness. To address this gap, we employed magnetron sputtering for precise control over AuNP deposition to enhance the photoluminescence of core–shell CdSe/CdS by using AuNPs. Moreover, we systematically investigated the effect of the morphology of AuNPs and spacer layer thickness on the PL enhancement.

## Experimental Methods

### Synthesis of CdSe/CdS Core–Shell Quantum Dots

#### Materials

All materials were purchased from Sigma-Aldrich unless otherwise stated and were used without any further purification. For the synthesis, the materials used were cadmium oxide (99.5%), cadmium acetate dihydrate (>98%), selenium powder (99.5+%), sulfur powder (>99%), oleic acid (90%), octadecene (90%), and trioctylphosphine (90%).

#### CdSe Quantum Dots Synthesis

Cadmium selenide QDs were synthesized using the hot-injection technique. 0.360 mmol of Se (28.5 mg) was mixed with 0.2 mL of TOP and 1.5 mL of ODE, and stirred for 20 min at 50 °C under argon atmosphere to obtain a transparent Se precursor. In parallel, 0.390 mmol of CdO (71 mg), 10 mL of ODE, and 1 mL of OA were mixed in a three-neck flask, evacuated on a Schlenk line, and heated to 280 °C for 20 min in an argon atmosphere until all the material was dissolved. At this point, the Se precursor was rapidly injected into the three-neck flask containing the Cd precursor, and the temperature was immediately lowered to 250 °C and maintained for 10 s to have controlled growth of CdSe quantum dots, followed by quenching to room temperature. The CdSe QDs were cleaned twice using methanol (1:3, v/v), centrifuged at 4000 rpm, and redispersed in octadecene for further use.

#### Growth of CdSe-CdS Core–Shell QD Structures

##### Preparation of Cd and S Precursors

Cadmium acetate dihydrate (54 mg, 0.2 mmol) and oleic acid (0.3 mL, 1 mmol) were added to 4.6 mL of 1-octadecene and were heated to 220 °C under stirring and an inert atmosphere for 10 min. Selenium powder (15.8 mg, 0.2 mmol) and tri-n-octylphosphine (0.1 mL, 0.4 mmol) were added to 4 mL of 1-octadecane and heated to 70 °C under an inert atmosphere until a clear mixture was obtained. Afterward, both precursor solutions were cooled to room temperature and stored for further use.

##### Growth of the CdS Shell around the CdSe Core

Shell growth was accomplished by one-pot shelling. In detail, 9 mL of 1-octadecene, CdSe cores (0.04 mmol), and calculated amounts of Cd and S precursors were added to a three-neck flask under an inert atmosphere and slowly heated to 200 °C. The temperature was stabilized at 200 °C to avoid triggering homogeneous nucleation, but it was sufficient for heterogeneous growth. The reaction was allowed to continue for 1 h before slowly cooling down to room temperature.

The CdSe-CdS core–shell materials were cleaned with methanol, centrifuged at 4000 rpm, and dispersed in hexane for further use.

### Fabrication of an AuNPs/QDs Coupling Structure

To demonstrate the PL enhancement of CdSe/CdS QDs by using AuNPs, different coupling structures were developed. The coupling structure with AuNPs deposited on a glass substrate followed by a spacer layer of PMMA and a top layer of CdSe/CdS QDs embedded in PMMA, as shown in Figure 1. First, gold nanoparticles (AuNPs) were deposited on a glass slide cleaned with ethanol and acetone. AuNPs were deposited by using magnetron sputtering. The glass samples were coated with carbon by using the pulsed carbon rod program on the sputter coater Quorum Q150T ES plus. Then, the gold nanoparticles were prepared on carbon-coated glass using the sputter coater Emitech K550X with a current of 10 mA for 10 s. The process is performed at a pressure of about 10^–1^ mbar in an argon atmosphere. After AuNPs, a PMMA spacer layer was prepared by spin coating at 3000 rpm for 60 s. The thickness was controlled by changing the concentration of the PMMA polymer in toluene. The thickness ranges from 3 to 51 nm for mass concentrations of 0.05, 0.075, 0.1, 0.3, 0.5, 0.7, 0.85, and 1.0 (wt %). The thickness of each concentration is listed in Figure 2. The method used by Song et al.^17^ was followed to reproduce the PMMA spacer layer to control the thickness. They measured the thickness of the PMMA layer using AFM. To make the top layer of QDs in PMMA, 1 mL of already prepared CdSe/CdS in toluene solution was taken and 1.0 wt % of PMMA in 1 mL of toluene was mixed. The solution after mixing was spin-coated as the top layer at 3000 rpm for 60 s. For each coupled structure, 5 different samples were made.

**Figure 1:**
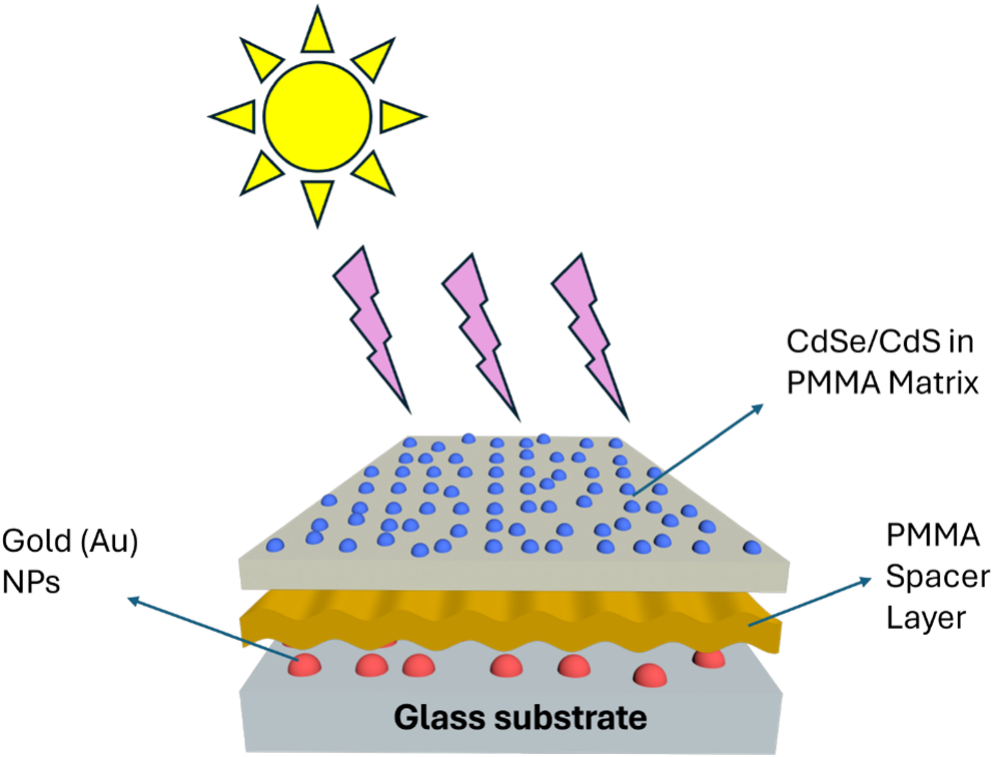
QDs and AuNPs coupled structure schematic (self-created).

**Figure 2:**
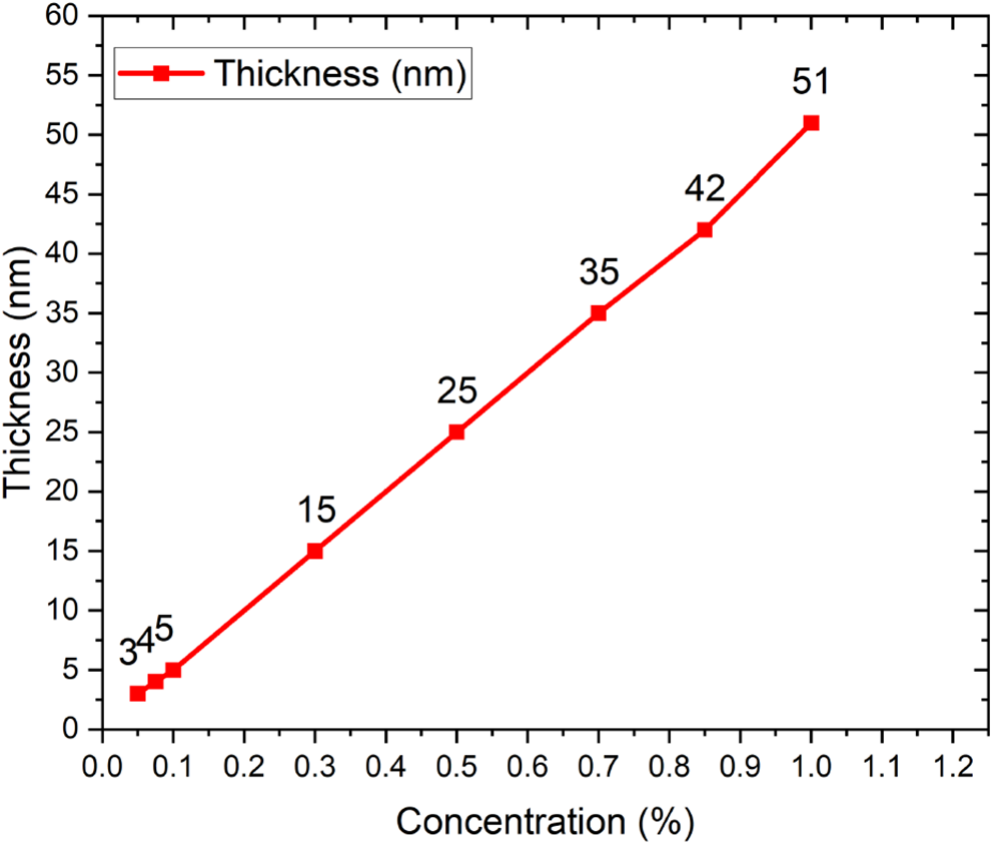
PMMA spacer layer thickness vs concentration (wt %).

### Optical Characterization

PL spectra of QDs were recorded by using an Optica PL spectrometer. The absorption spectra were recorded by using a Cary 60 UV–vis spectrometer. PL spectra of the coupling were recorded using a Renishaw inVia confocal Raman microscope (Model: Inspect). The laser was replaced by an external UV LED. The excitation wavelength of the source was 375 nm (approx: ±10 nm). Measurements were performed by using a 2400 l/mm diffractive grating. The spectra were acquired by a 576-pixel CCD detector. The UV lamp was positioned at a distance of 10 cm and maintained at the same angle. A 100× objective lens was used to collect the emitted PL signal. All of the readings were taken in a dark room at room temperature.

## Results and Discussion

### Morphology of Gold Nanoparticles (AuNPs)

To investigate the morphology of AuNPs deposited, scanning electron microscopy (SEM) was used. The samples have been analyzed using the Zeiss Gemini300 scanning electron microscope, working with an acceleration voltage of 5 kV, at a working distance of 5 mm, and acquiring both the secondary electrons (with the InLens detector) and the backscattered electrons with the external BSD detectorTo get the baseline reference for AuNPs deposition, bare glass slide was coated with carbon as shown in Figure 3. The reason for carbon coating as it was not detectable by SEM without it. However, for the PL of the coupled structure, it was not coated with carbon. The brighter white spots in Figure 4 show spherical-shaped AuNPs well dispersed on the glass substrate with minimal clustering or agglomeration. To determine the size of the AuNPs, ImageJ software was used.

**Figure 3:**
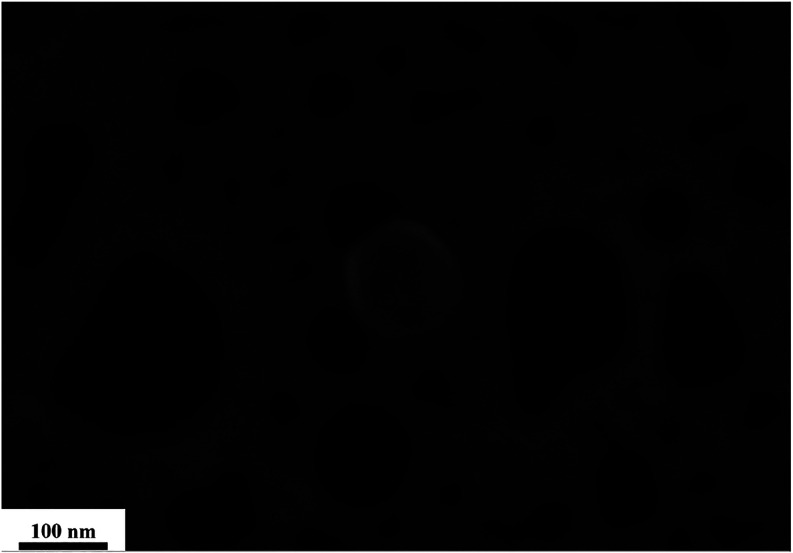
SEM image of bare glass coated with carbon.

**Figure 4:**
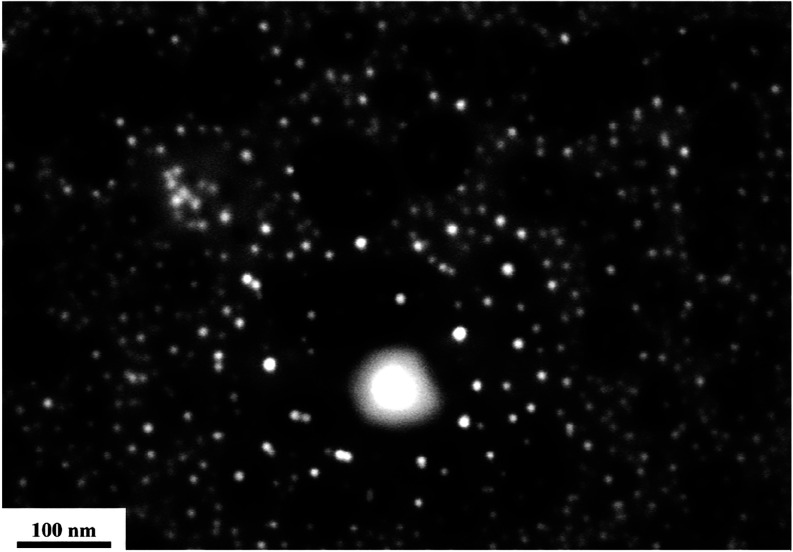
SEM image of AuNPs on glass coated with carbon.

Figure 5 illustrates the particle size distribution of the nanoparticles. The particle distribution is in the narrow range 6 to 18 with an average size of 11.1 nm. Primarily, the size of the NPs lies in the range 9 to 12 nm. Such a uniform distribution of size is essential for achieving a consistent localized surface plasmon resonance. The uniform distribution of nanoparticle shape and size plays a crucial role in achieving PL enhancement of QDs through LSPR.^30^

**Figure 5:**
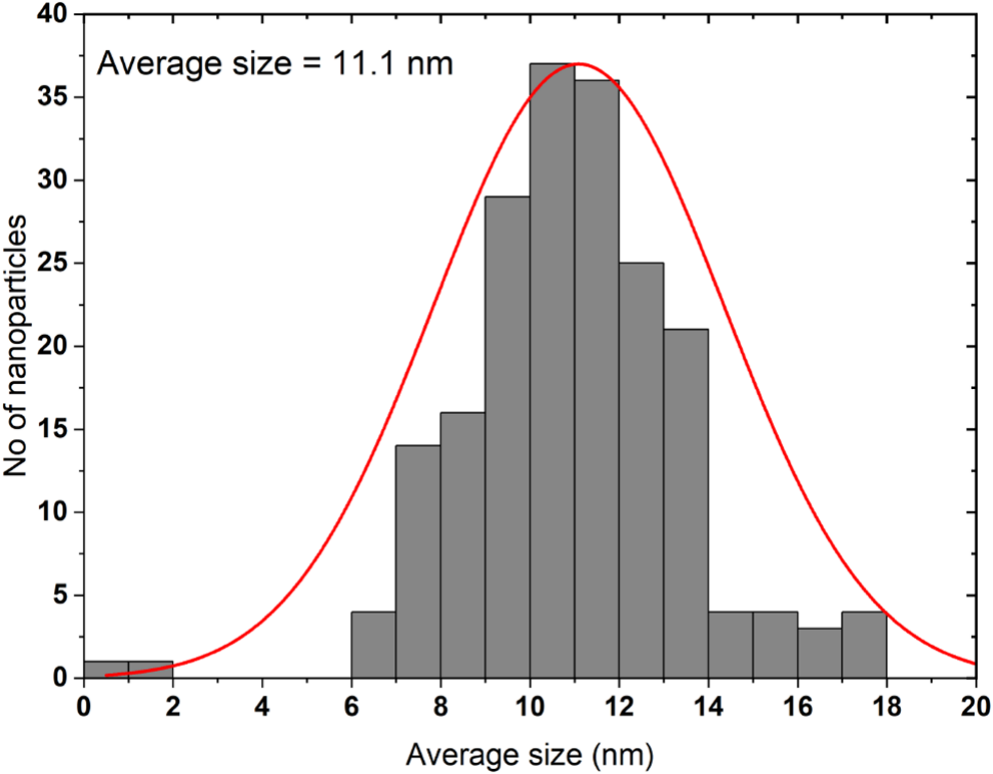
AuNPs size distribution.

### Optical Properties of AuNPs and CdSe/CdS QDs

The absorption spectra of AuNPs deposited at 10 mA for varying duration, starting from 10 to 50 s, are shown in Figure 6. The spectra exhibit clear surface plasmon resonance peaks located in the visible region, ranging from 520 to 595 nm, which increase as the deposition time increases. This confirms successful nanoparticle formation and consistent size control, which is the expected optical behavior for such a configuration and makes it suitable for LSPR applications.^31,32^

**Figure 6:**
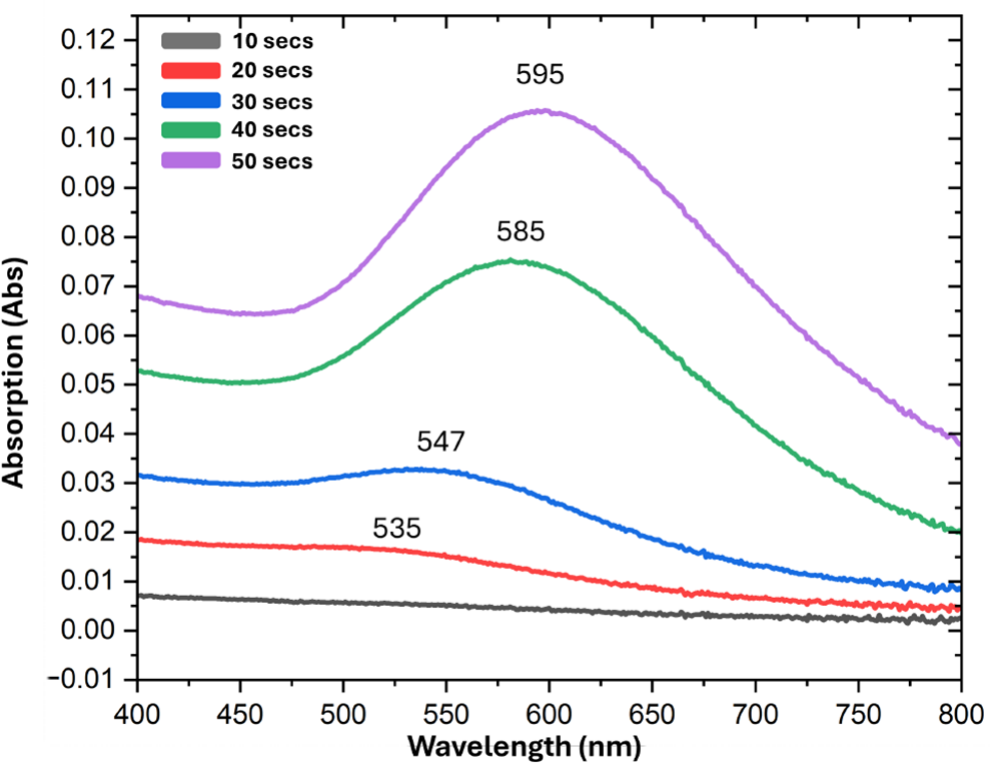
Absorption spectra of AuNPs sputtered at 10 mA for different periods of time.

Figure 7 shows that the CdSe core quantum dots exhibited an absorption peak at 515 nm, corresponding to a core diameter of approximately 2.6 nm, as estimated using the method described by Peng et al.^33^ A single CdS shell layer was subsequently grown on the CdSe cores by using the successive ionic layer adsorption and reaction (SILAR) technique. Based on a previously determined shell growth rate of 0.7 nm per hour, the final size of the resulting CdSe–CdS core–shell quantum dots was estimated to be approximately 3.3 nm.

**Figure 7:**
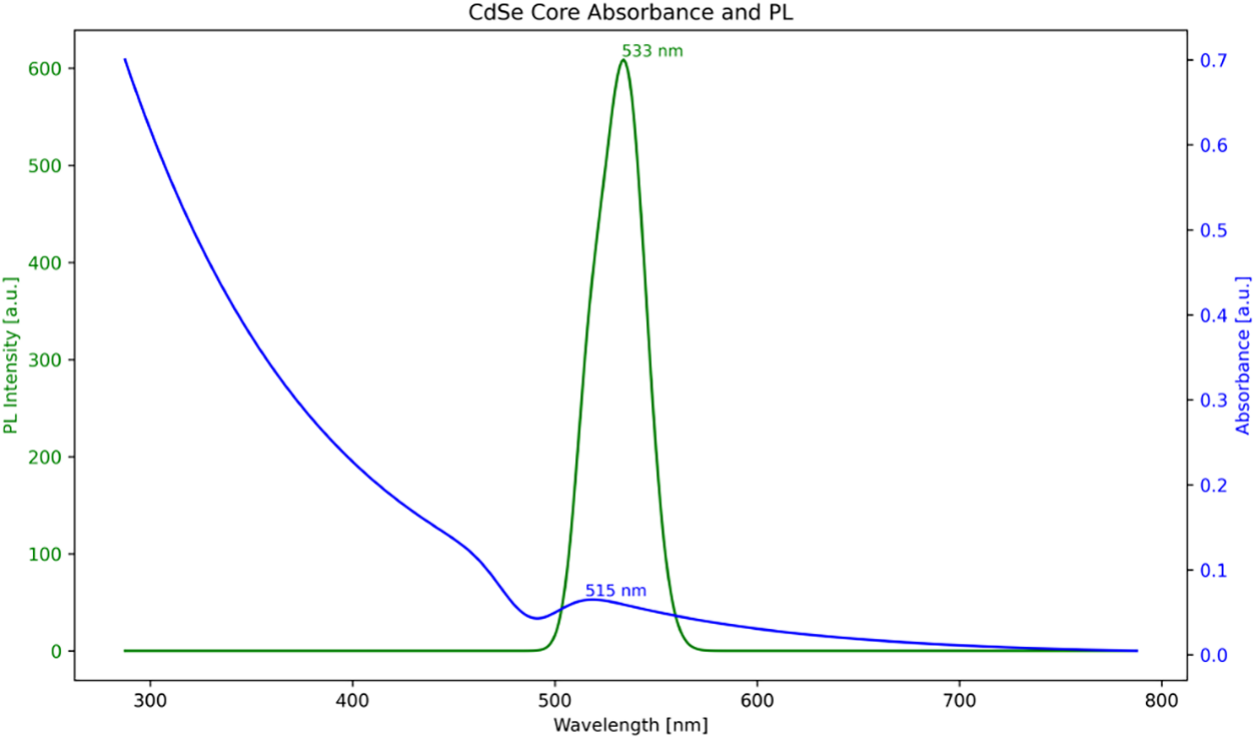
Absorption and photoluminescence spectra of the CdSe core.

Figure 8 shows the photoluminescence (PL) and absorption spectra of CdSe/CdS core–shell QDs. The absorption spectrum indicates strong absorption in the UV region, representing efficient utilization of high-energy photons. The PL emission spectrum shows a pronounced emission peak in the visible region. To get the effective LSPR coupling between metal nanoparticles and the QDs, the absorption spectra of surface plasmons AuNPs should overlap with the PL emission of gold nanoparticles.^34^

**Figure 8:**
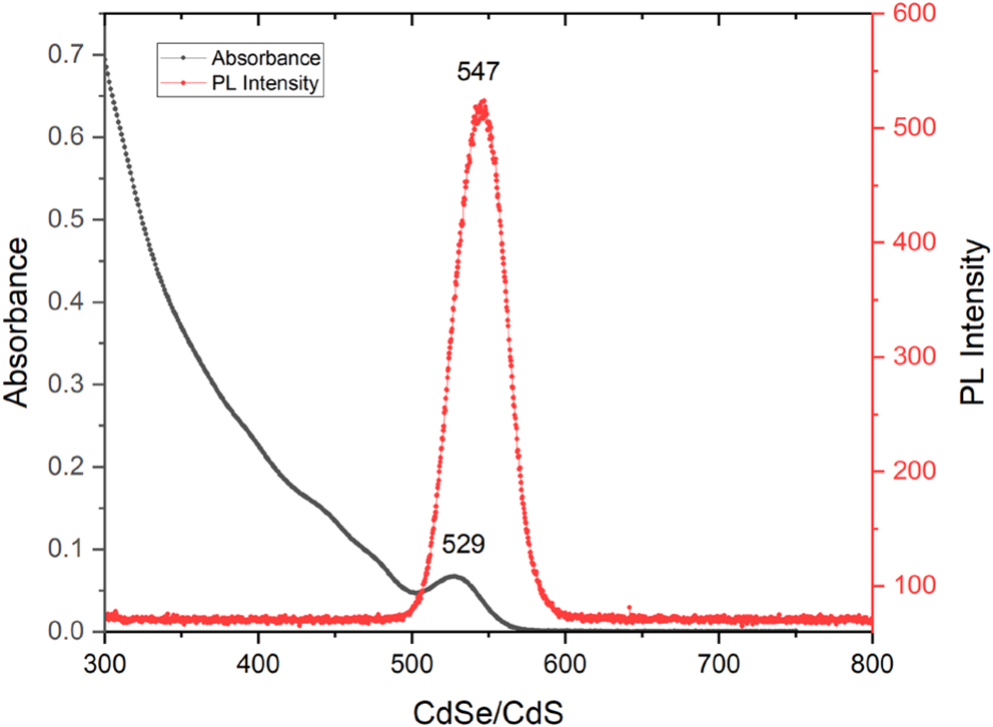
Absorption and photoluminescence spectra of CdSe/CdS.

The observed characteristics explained in this section demonstrate the feasibility of the PL enhancement of CdSe/CdS quantum dots using AuNPs. The optimal resonance absorption and size uniformity are essential for amplifying the localized electric fields, resulting in stronger interactions and energy transfer to the QDs. The PL enhancement is not multifold as the energy transfer is minimal due to the spectral mismatch between the excitation source and the AuNPs absorption peak. This limits the direct resonant-mediated energy transfer. However, considering the absorption spectra of AuNPs, it has a peak at 585 nm, but they do absorb at lower wavelengths as well, extending into the UV–visible region. The reason is that a 375 nm excitation source is used to investigate how QDs and AuNPs coupled structures respond to UV light. Moreover, the aim is to examine these QDs and AuNPs coupled structures as potential UV downshifting layers and whether their near-field enhancement could still modulate QD emission. Though the plasmon lifetimes are indeed short, this coupling interaction enhances the QDs’ excitation rate primarily due to the local field enhancement and not purely resonant energy transfer.^35^

**Figure 9:**
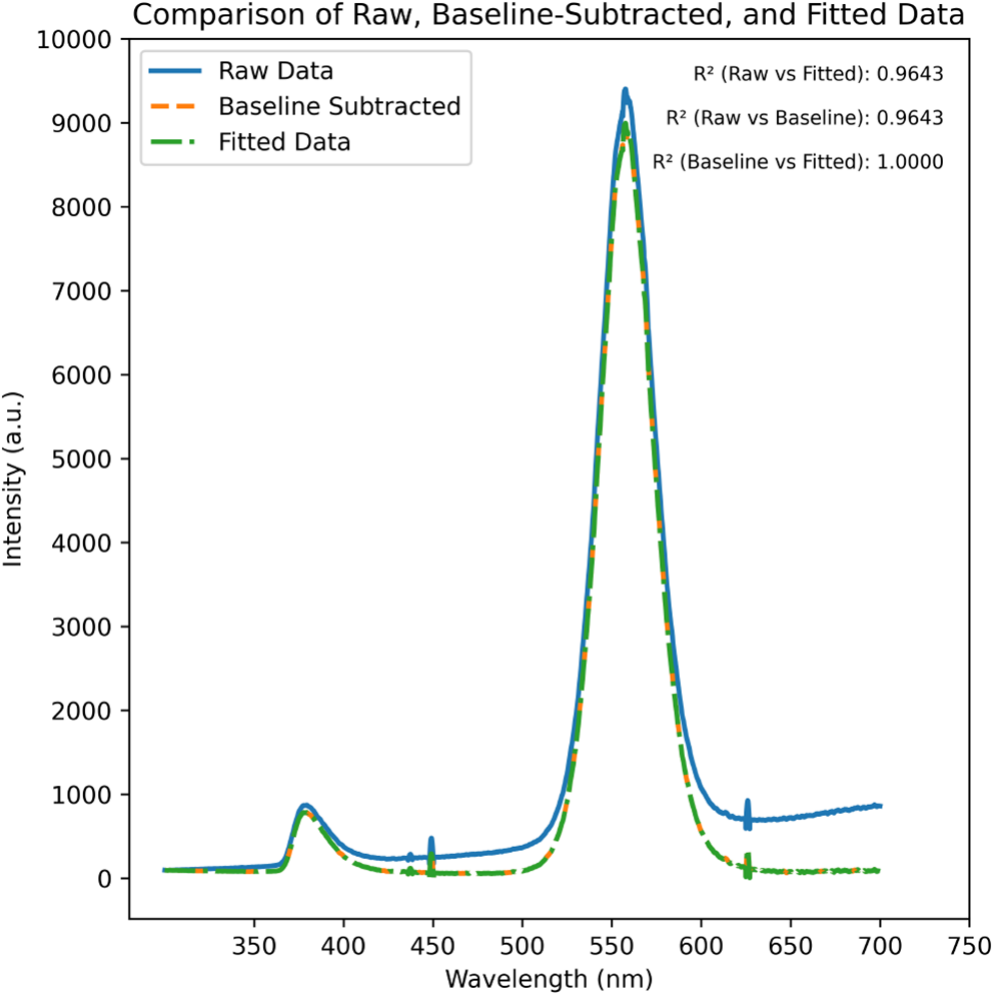
Sample plot showing baseline subtraction and peak fitting with *R*-square values.

### Plasmonic Effect of QDs and AuNPs Coupling Structure

Raw PL data for different configurations of the coupling structures, starting from the scenario with no spacer layer to a progressively thicker PMMA spacer layer, are given in Figures 10 and 11. Before normalization of the data, it was preprocessed to remove anomalies by subtracting the baseline and fitting the two distinct peaks, as shown in Figure 9. The baseline subtraction and peak fitting were done using Fityk software (open source). The Figure from Fityk GUI has been added in the Supporting Information file as Figure 3. A large emission peak (corresponding to the QDs’ emission around 550 nm) was fitted using the pseudo-Voigt function. This function suitably accounts for both Gaussian and Lorentzian line shapes, typical of semiconductor nanocrystal emission spectra. Similarly, the smaller peak at around 375 nm was fitted using a log-normal function, typically appropriate for narrower and asymmetrical spectral features. This peak fitting was done to acquire the area under the curve for the two distinct peaks. To understand the scenario better, the sample plot after data preprocessing is given in Figure 8 for 15 nm thicker PMMA spacer layer-coupled structure of QDs and AuNPs. The *R*-square values of 1.0 and 0.96, as depicted in Figure 9, show that both the functions selected for peak fitting have almost perfectly fitted. To remove the noises from the raw data, it has been smoothed using the Savitzky–Golay smoothing filter in Origin Pro software. However, this is not a true representation of PL enhancement from the raw PL data. To infer the true picture from the raw PL data, it was normalized. The reason for normalization is to eliminate the influence of the scattering effect of the excitation source on the PL of QDs and AuNPs coupled structure, if it exists. As is evident from Figure 11(left), there is no direct relationship between the peak of the excitation source and the coupled structure. If there exists any dependency between the change in the peak height of the excitation source and the coupled structure geometry, it would be understandable that the coupled structure geometry causes scattering phenomena. The data is normalized by taking the ratio of the area under the curve for both the PL peak and the peak of the excitation source. The ratio-based normalization is used to compare relative enhancement across samples and does not reflect the quantification of absolute emission. The area under the curve for two peaks was determined using Fityk software, as shown in Figure 3 in the Supporting Information file. The result of the 15 nm spacer layer-coupled structure is given in Table 1.

**Figure 10:**
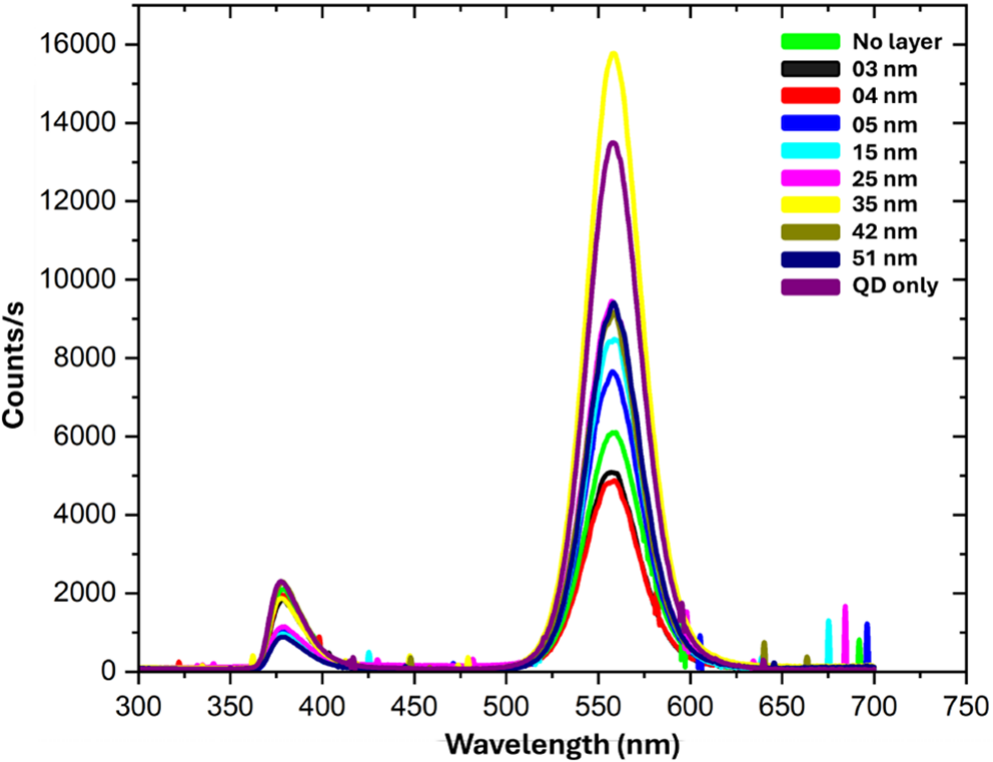
To remove the noise from the raw data, it has been smoothed using the Savitzky–Golay smoothing filter in Origin Pro software.

**Figure 11:**
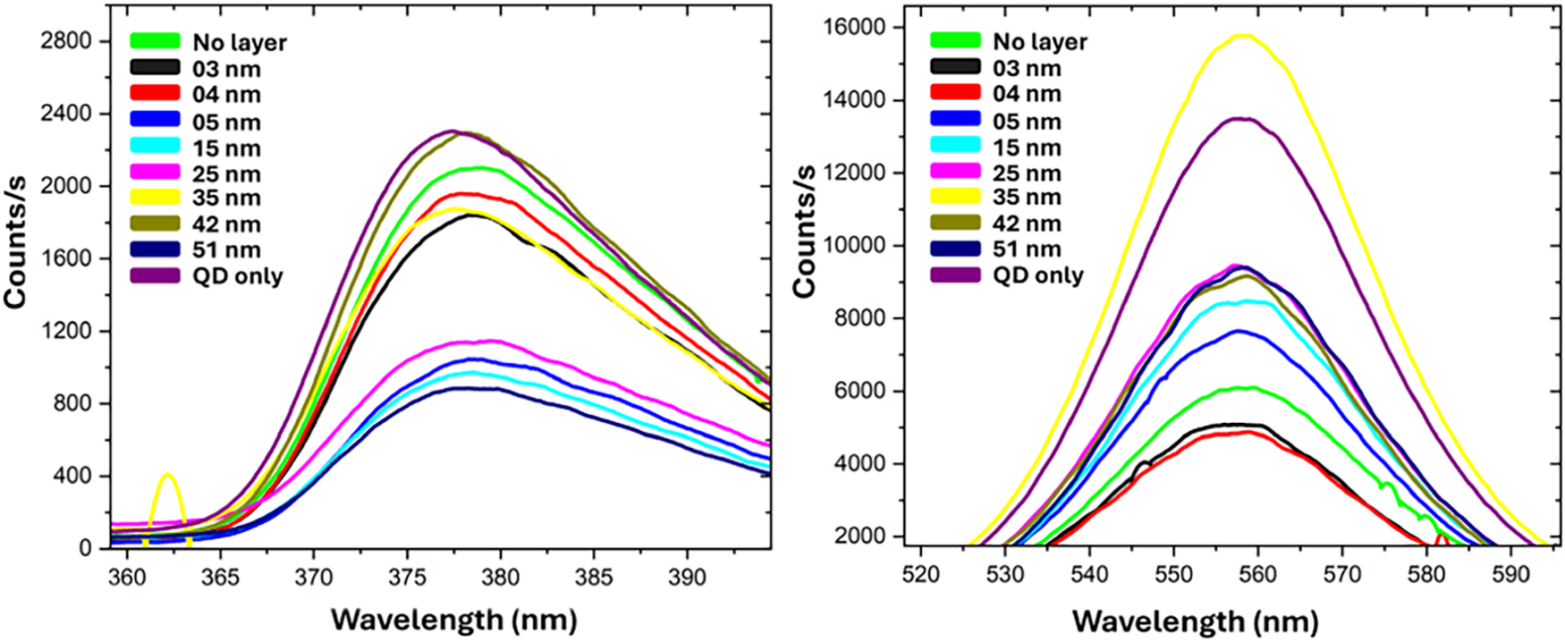
Enlarged image of the excitation peak (left) and PL emission peak (right) of the plasmonic coupling structure.

**Table 1:** Area under the Curves and Its Ratio

sample name	peak of excitation source (area under the curve) (function: log normal)	peak of QDs emission (area under the curve) (function: Lorentzian)	area ratio
sample (a)^a^, 15 nm thickness	20,653.6	366,322	17.43

a This refers to a specific sample with 15 nm thickness.

Figure 12 demonstrates the normalized photoluminescence PL data through box plots of the five different samples prepared using the same method as that explained earlier in the methodology section. The normalization of data was done by calculating the ratio of areas of the two distinctive peaks in the raw PL data. Subsequently, the normalized PL data is obtained by taking the ratio of the area under the larger emission peak and the smaller excitation peak for each coupling configuration. From the Normalized PL data as depicted in Figure 11, the PL of AuNPs and QDs coupling structure with no spacer layer (direct contact) is significantly lower (average ≈ 6) than the QD PL (average ≈ 10). Similarly, for lower concentration 0.05 to 0.075 wt % (corresponding spacer layer thickness of 3–4 nm), the normalized PL (average ≈ 5) is almost half for the QDs only. This lower ratio is an indication of pronounced quenching and consequently nonradiative energy transfer, due to the closer proximity of QDs to AuNPs.^36–38^ Significant PL enhancement (average ≈ 13) is first observed for the concentration of PMMA of 0.1 wt % (corresponds to 5 nm thickness of spacer layer). The average normalized PL increases from 13 to 16 for the spacer layer thickness starting from 5 nm, reaching a maximum at a thickness of 42 nm. At 0.5 wt % concentration (thickness-25 nm), the average normalized PL was 16.3, the highest as compared to QDs only (average ≈ 10). This reflects an optimized condition for maximum QD emissions to the relative excitation input, indicating strong plasmonic enhancement of the radiative emission process.^39,40^ However, further increasing PMMA thickness beyond 42 nm causes a reduction in normalized PL. This reduction in PL highlights the diminishing plasmonic effect, which is almost similar to that of the coupling structure without a spacer layer. This demonstrates the importance of spacer layer thickness to exploit the plasmonic effects for PL enhancement of QDs.^41,42^

**Figure 12:**
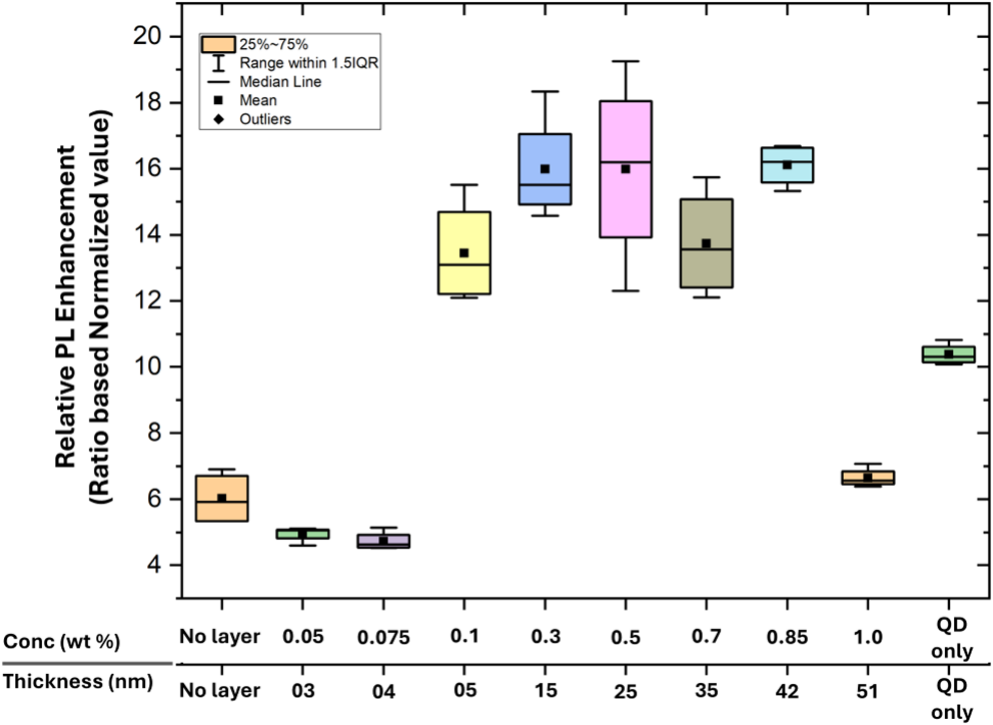
Box plot of normalized PL data of the plasmonic coupling structure.

## Conclusions

This study focuses on PL enhancement of CdSe/CdS core–shell QDs using gold nanoparticles through optimized plasmonic interactions. The magnetron sputtering deposition method was used for AuNPs deposition. This paves the way for the scalability of this coupling approach for PL enhancement. Substantial PL enhancement was achieved by precisely controlling the morphology and fine-tuning the PMMA spacer layer thickness. The maximum PL enhancement was achieved at a spacer layer thickness of 25 nm. Excessive closeness, i.e., below 5 nm, results in quenching due to nonradiative energy transfer, whereas large separation beyond 42 nm causes diminishing plasmonic effects. The optimal configuration of the CdSe/CdS and AuNPs coupling structure confirms the potential for substantial performance improvement in solar cell performance. Future research should further explore other coupling mechanisms and their integration into solar cell technologies to expand their applicability.

## Supplementary Material



## Notes

Raw PL data of the QDs and AuNPs coupling structure (ZIP)

## References

[1] Chen M., Lu L., Yu H., Li C., Zhao N. (2021). Integration of Colloidal
Quantum Dots with Photonic Structures for Optoelectronic and Optical
Devices. Adv. Sci..

[2] Chen O., Zhao J., Chauhan V. P., Cui J., Wong C., Harris D. K., Wei H., Han H.-S., Fukumura D., Jain R. K., Bawendi M. G. (2013). Compact high-quality
CdSe-CdS core-shell
nanocrystals with narrow emission linewidths and suppressed blinking. Nat. Mater..

[3] Wood V., Bulović V. (2010). Colloidal quantum dot light-emitting devices. Nano Rev..

[4] Abdullah M. M., Ibrahim O. A. (2020). CdSe/CdS Quantum Dot Core-Shell for Silicon Solar Cell
Efficiency Enhancement. Nano Hybrids Compos..

[5] Lopez-Delgado R., Zhou Y., Zazueta-Raynaud A., Zhao H., Pelayo J. E., Vomiero A., Ramos M. E. Á., Rosei F., Ayon A. (2017). Enhanced conversion
efficiency in Si solar cells employing photoluminescent down-shifting
CdSe/CdS core/shell quantum dots. Sci. Rep..

[6] Werschler F., Lindner B., Hinz C., Conradt F., Gumbsheimer P., Behovits Y., Negele C., de Roo T., Tzang O., Mecking S., Leitenstorfer A., Seletskiy D. V. (2018). Efficient
Emission Enhancement of Single CdSe/CdS/PMMA Quantum Dots through
Controlled Near-Field Coupling to Plasmonic Bullseye Resonators. Nano Lett..

[7] Kim I., Moon J.-S., Kyhm K., Oh J.-W. (2017). Surface plasmon-assisted
photoluminescence enhancement of Au-hybrid CdSe/ZnS nanocrystal quantum
dots. Mol. Cryst. Liq. Cryst..

[8] Subhan A., Mourad A.-H. I. (2025). Plasmonic metal
nanostructures as performance enhancers
in emerging solar cells: A review. Next Mater..

[9] Fu W.-F., Chen X., Yang X., Wang L., Shi Y., Shi M., Li H.-Y., Jen A. K.-Y., Chen J.-W., Cao Y., Chen H.-Z. (2013). Optical
and electrical effects of plasmonic nanoparticles
in high-efficiency hybrid solar cells. Phys.
Chem. Chem. Phys..

[10] Yang Y., Dev A., Sychugov I., Hägglund C., Zhang S.-L. (2023). Plasmon-Enhanced
Fluorescence of Single Quantum Dots Immobilized in Optically Coupled
Aluminum Nanoholes. J. Phys. Chem. Lett..

[11] Cao J., Zhang H., Pi X., Li D., Yang D. (2021). Enhanced photoluminescence
of silicon quantum dots in the presence of both energy transfer enhancement
and emission enhancement mechanisms assisted by the double plasmon
modes of gold nanorods. Nanoscale Adv..

[12] Kroychuk M. K., Shorokhov A. S., Yagudin D. F., Rakhlin M. V., Klimko G. V., Toropov A. A., Shubina T. V., Fedyanin A. A. (2023). Quantum Dot Photoluminescence
Enhancement in GaAs Nanopillar Oligomers Driven by Collective Magnetic
Modes. Nanomaterials.

[13] Meixner A. J., Jäger R., Jäger S., Bräuer A., Scherzinger K., Fulmes J., Krockhaus S. z. O., Gollmer D. A., Kern D. P., Fleischer M. (2015). Coupling single
quantum dots to plasmonic nanocones: optical properties. Faraday Discuss..

[14] Kumar V., Nisika N., Kumar M. (2020). Modified Absorption and Emission
Properties Leading to Intriguing Applications in Plasmonic-Excitonic
Nanostructures. Adv. Opt. Mater..

[15] Samanta A., Zhou Y., Zou S., Yan H., Liu Y. (2014). Fluorescence
Quenching of Quantum Dots by Gold Nanoparticles: A Potential Long
Range Spectroscopic Ruler. Nano Lett..

[16] Goryacheva, O. A. ; Beloglazova, N. ; De Saeger, S. ; Goryacheva, I. Y. In Quantum Dots—Gold Nanoparticles FRET Based System Immunoassay, 2018 International Conference Laser Optics (ICLO); IEEE, 2018; pp 403.

[17] Song M., Wu B., Chen G., Liu Y., Ci X., Wu E., Zeng H. (2014). Photoluminescence Plasmonic Enhancement
of Single Quantum Dots Coupled
to Gold Microplates. J. Phys. Chem. C.

[18] Okamoto T., Onishi A., Shi X., Oshikiri T., Ueno K., Misawa H., Biju V. (2024). Distance-Dependent
Energy Transfer
under Modal Strong Coupling from CdSe/ZnS Quantum Dots to a Plasmonic
Fabry-Pérot Cavity. J. Phys. Chem. C.

[19] Hu L., Xu T., Zhu H., Ma C., Chen G. (2019). Luminescence Change
of CdS and CdSe Quantum Dots on a Ag Film. ACS
Omega.

[20] Cirillo M., Aubert T., Gomes R., Van Deun R., Emplit P., Biermann A., Lange H., Thomsen C., Brainis E., Hens Z. (2014). “Flash” Synthesis of CdSe/CdS Core-Shell Quantum Dots. Chem. Mater..

[21] Xing W., Zhang S., An R., Bi W., Geng C., Xu S. (2021). Low-temperature synthesis of tetrapod CdSe/CdS quantum dots through
a microfluidic reactor. Nanoscale.

[22] Mukherjee D., Kertész K., Zolnai Z., Kovács Z., Deák A., Pálinkás A., Osváth Z., Olasz D., Romanenko A., Fried M., Burger S., Sáfrán G., Petrik P. (2025). Optimized sensing on gold nanoparticles
created by graded-layer magnetron sputtering and annealing. Sens. Actuators, B.

[23] Liu S., Li X., Hao Y., Li X., Liu F. (2023). Effect of
magnetron
sputtering process parameters on the conductivity of thin metal film. AIP Adv..

[24] Bulut Y., Sochor B., Reck K. A., Schummer B., Meinhardt A., Drewes J., Liang S., Guan T., Jeromin A., Stierle A., Keller T. F., Strunskus T., Faupel F., Müller-Buschbaum P., Roth S. V. (2024). Investigating
Gold Deposition with High-Power Impulse Magnetron Sputtering and Direct-Current
Magnetron Sputtering on Polystyrene, Poly-4-vinylpyridine, and Polystyrene
Sulfonic Acid. Langmuir.

[25] Bhardwaj N., Satpati B., Mohapatra S. (2020). Plasmon-enhanced
photoluminescence
from SnO2 nanostructures decorated with Au nanoparticles. Appl. Surf. Sci..

[26] Wang H., Xu L., Zhang R., Ge Z., Zhang W., Xu J., Ma Z., Chen K. (2015). Controllable
photoluminescence enhancement of CdTe/CdS
quantum dots thin films incorporation with Au nanoparticles. Nanoscale Res. Lett..

[27] Perveen A., Zhang X., Tang J.-L., Han D.-B., Chang S., Deng L.-G., Ji W.-Y., Zhong H.-Z. (2018). Sputtered gold nanoparticles
enhanced quantum dot light-emitting diodes. Chin. Phys. B.

[28] Shu Q., Huang P., Yang F., Yang L., Chen L. (2023). Study on crystal
growth of Ge/Si quantum dots at different Ge deposition by using magnetron
sputtering technique. Sci. Rep..

[29] Nguyen H. T., Tran T. T., Bhatt V., Kumar M., Yun J.-H. (2022). Photoluminescence
Properties of CdSe/ZnS Quantum Dot Donor-Acceptor via Plasmon Coupling
of Metal Nanostructures and Application on Photovoltaic Devices. J. Phys. Chem. Lett..

[30] Depciuch J., Stec M., Maximienko A., Baran J., Parlinska-Wojtan M. (2022). Size-dependent
theoretical and experimental photothermal conversion efficiency of
spherical gold nanoparticles. Photodiagn. Photodyn.
Ther..

[31] Zhai Y., Wang Q., Qi Z., Li C., Xia J., Li X. (2017). Experimental investigation of energy
transfer between CdSe/ZnS quantum
dots and different-sized gold nanoparticles. Phys. E.

[32] West R., Sadeghi S. M. (2012). Enhancement of Energy Transfer between Quantum Dots:
The Impact of Metallic Nanoparticle Sizes. J.
Phys. Chem. C.

[33] Yu W. W., Qu L., Guo W., Peng X. (2003). Experimental Determination of the
Extinction Coefficient of CdTe, CdSe, and CdS Nanocrystals. Chem. Mater..

[34] Kim N.-Y., Hong S.-H., Kang J.-W., Myoung N., Yim S.-Y., Jung S., Lee K., Tu C. W., Park S.-J. (2015). Localized
surface plasmon-enhanced green quantum dot light-emitting diodes using
gold nanoparticles. RSC Adv..

[35] Milekhin I. A., Anikin K. V., Rahaman M., Rodyakina E. E., Duda T. A., Saidzhonov B. M., Vasiliev R. B., Dzhagan V. M., Milekhin A. G., Batsanov S. A., Gutakovskii A. K., Latyshev A. V., Zahn D. R. T. (2020). Resonant plasmon
enhancement of light
emission from CdSe/CdS nanoplatelets on Au nanodisk arrays. J. Chem. Phys..

[36] Hildebrandt N., Lim M., Kim N., Choi D. Y., Nam J.-M. (2023). Plasmonic quenching
and enhancement: metal-quantum dot nanohybrids for fluorescence biosensing. Chem. Commun..

[37] Li J.-Y., Li W., Liu J., Zhong J., Liu R., Chen H., Wang X.-H. (2022). Room-Temperature Strong Coupling Between a Single Quantum
Dot and a Single Plasmonic Nanoparticle. Nano
Lett..

[38] Dikmen Z. (2024). Gold-tipped
CdSe/CdZnS colloidal quantum wells as non-quenching plasmonic particles
for optical applications. Opt. Mater..

[39] Singh S., Raulo A., Singh A., Mittal M., Horechyy A., Hübner R., Formanek P., Srivastava R. K., Sapra S., Fery A., Nandan B. (2021). Enhanced Photoluminescence
of Gold Nanoparticle-Quantum Dot Hybrids Confined in Hairy Polymer
Nanofibers. ChemNanoMat.

[40] Pan J., Chen J., Zhao D., Huang Q., Khan Q., Liu X., Tao Z., Zhang Z., Lei W. (2016). Surface plasmon-enhanced
quantum dot light-emitting diodes by incorporating gold nanoparticles. Opt. Express.

[41] Xiong Y., Zhang X. (2019). InAs/InP quantum dots stacking: Impact of spacer layer on optical
properties. J. Appl. Phys..

[42] Mendes M. J., Hernández E., López E., García-Linares P., Ramiro I., Artacho I., Antolín E., Tobías I., Martí A., Luque A. (2013). Self-organized colloidal
quantum dots and metal nanoparticles for plasmon-enhanced intermediate-band
solar cells. Nanotechnology.

