# Supplementary material for: Photoluminescence Enhancement in CdSe/CdS Quantum Dot Colloidal Films Induced by Gold Nanoparticles (AuNPs)
Source: ACS Omega. 2025 Sep 8;10(37):42472–9. doi: 10.1021/acsomega.5c03718 (PMC12461293; doi:10.1021/acsomega.5c03718)
Supplement: Supplementary file 1 [file ao5c03718_si_001.zip › achemso-demo.pdf]

A demonstration of the achemso $\LaTeX$ class[†]

Andrew N. Other,[‡,∥] Fred T. Secondauthor,[‡,⊥] I. Ken Groupleader,[*,‡,¶,∥] Susanne K. Laborator,[*,§] and Kay T. Finally[‡,¶]

‡*Department of Chemistry, Unknown University, Unknown Town*

¶*Department of Chemistry, Second University, Nearby Town*

§*Lead Discovery, BigPharma, Big Town, USA*

∥*A shared footnote*

⊥*Current address: Some other place, Othertöwn, Germany*

E-mail: i.k.groupleader@unknown.uu; s.k.laborator@bigpharma.co

Phone: +123 (0)123 4445556. Fax: +123 (0)123 4445557

Abstract

This is an example document for the achemso document class, intended for submissions to the American Chemical Society for publication. The class is based on the standard $\LaTeX\,2_\varepsilon$ report file, and does not seek to reproduce the appearance of a published paper.

This is an abstract for the achemso document class demonstration document. An abstract is only allowed for certain manuscript types. The selection of `journal` and `manuscript` will determine if an abstract is valid. If not, the class will issue an appropriate error.

[†]A footnote for the title

Introduction

This is a paragraph of text to fill the introduction of the demonstration file. The demonstration file attempts to show the modifications of the standard LaTeX macros that are implemented by the achemso class. These are mainly concerned with content, as opposed to appearance.

Results and discussion

Outline

The document layout should follow the style of the journal concerned. Where appropriate, sections and subsections should be added in the normal way. If the class options are set correctly, warnings will be given if these should not be present.

References

The class makes various changes to the way that references are handled. The class loads natbib, and also the appropriate bibliography style. References can be made using the normal method; the citation should be placed before any punctuation, as the class will move it if using a superscript citation style.[1–4] The use of natbib allows the use of the various citation commands of that package: Abernethy et al. have shown something, in 1999, or as given by Ref. 1. Long lists of authors will be automatically truncated in most article formats, but not in supplementary information or reviews.[6] If you encounter problems with the citation macros, please check that your copy of natbib is up to date. The demonstration database file `achemso-demo.bib` shows how to complete entries correctly. Notice that "et al." is auto-formatted using the `\latin` command.

Multiple citations to be combined into a list can be given as a single citation. This uses the mciteplus package.[7] Citations other than the first of the list should be indicated with

a star. If the mciteplus package is not installed, the standard bibliography tools will still work but starred references will be ignored. Individual references can be referred to using `\mciteSubRef`: "ref. 7.c".

The class also handles notes to be added to the bibliography. These should be given in place in the document.[8] As with citations, the text should be placed before punctuation. A note is also generated if a citation has an optional note. This assumes that the whole work has already been cited: odd numbering will result if this is not the case.[9]

Floats

New float types are automatically set up by the class file. The means graphics are included as follows (Scheme 1). As illustrated, the float is "here" if possible.

Your scheme graphic would go here: `.eps` format
for LaTeX or `.pdf` (or `.png`) for pdfLaTeX
ChemDraw files are best saved as `.eps` files:
these can be scaled without loss of quality, and can be
converted to `.pdf` files easily using `eps2pdf`.

Scheme 1: An example scheme

As well as the standard float types `table`
and `figure`, the class also recognises
`scheme`, `chart` and `graph`.

Figure 1: An example figure

Charts, figures and schemes do not necessarily have to be labelled or captioned. However, tables should always have a title. It is possible to include a number and label for a graphic without any title, using an empty argument to the `\caption` macro.

The use of the different floating environments is not required, but it is intended to make document preparation easier for authors. In general, you should place your graphics where they make logical sense; the production process will move them if needed.

Math(s)

The achemso class does not load any particular additional support for mathematics. If packages such as amsmath are required, they should be loaded in the preamble. However, the basic LaTeX math(s) input should work correctly without this. Some inline material $y = mx + c$ or $1 + 1 = 2$ followed by some display.

$$A = \pi r^2$$

It is possible to label equations in the usual way (Eq. 1).

$$\frac{\mathrm{d}}{\mathrm{d}x} r^2 = 2r \tag{1}$$

This can also be used to have equations containing graphical content. To align the equation number with the middle of the graphic, rather than the bottom, a minipage may be used.

As illustrated here, the width of the minipage needs to allow some space for the number to fit in to. (2)

Experimental

The usual experimental details should appear here. This could include a table, which can be referenced as Table 1. Notice that the caption is positioned at the top of the table.

Table 1: An example table

Header one	Header two
Entry one	Entry two
Entry three	Entry four
Entry five	Entry five
Entry seven	Entry eight

Adding notes to tables can be complicated. Perhaps the easiest method is to generate

these using the basic \textsuperscript and \emph macros, as illustrated (Table 2).

Table 2: A table with notes

Header one	Header two
Entry one[a]	Entry two
Entry three[b]	Entry four

[a] Some text; [b] Some more text.

The example file also loads the optional mhchem package, so that formulas are easy to input: \ch{H2SO4} gives H_2SO_4. See the use in the bibliography file (when using titles in the references section).

The use of new commands should be limited to simple things which will not interfere with the production process. For example, \mycommand has been defined in this example, to give italic, mono-spaced text: *some text*.

Extra information when writing JACS Communications

When producing communications for *J. Am. Chem. Soc.*, the class will automatically lay the text out in the style of the journal. This gives a guide to the length of text that can be accommodated in such a publication. There are some points to bear in mind when preparing a JACS Communication in this way. The layout produced here is a *model* for the published result, and the outcome should be taken as a *guide* to the final length. The spacing and sizing of graphical content is an area where there is some flexibility in the process. You should not worry about the space before and after graphics, which is set to give a guide to the published size. This is very dependant on the final published layout.

You should be able to use the same source to produce a JACS Communication and a normal article. For example, this demonstration file will work with both type=article and type=communication. Sections and any abstract are automatically ignored, although you will get warnings to this effect.

Acknowledgement

Please use "The authors thank ..." rather than "The authors would like to thank ...".

The author thanks Mats Dahlgren for version one of achemso, and Donald Arseneau for the code taken from cite to move citations after punctuation. Many users have provided feedback on the class, which is reflected in all of the different demonstrations shown in this document.

Supporting Information Available

A listing of the contents of each file supplied as Supporting Information should be included. For instructions on what should be included in the Supporting Information as well as how to prepare this material for publications, refer to the journal's Instructions for Authors.

The following files are available free of charge.

- Filename: brief description
- Filename: brief description

References

(1) Abarca, A.; Gómez-Sal, P.; Martín, A.; Mena, M.; Poblet, J. M.; Yélamos, C. Ammonolysis of mono(pentamethylcyclopentadienyl) titanium(IV) derivatives. *Inorg. Chem.* **2000**, *39*, 642–651.

(2) Abernethy, C. D.; Codd, G. M.; Spicer, M. D.; Taylor, M. K. A highly stable N-heterocyclic carbene complex of trichloro-oxo-vanadium(V) displaying novel Cl—C(carbene) bonding interactions. *J. Am. Chem. Soc.* **2003**, *125*, 1128–1129.

(3) Friedman-Hill, E. *Jess in Action: Java Rule-based Systems*, 1st ed.; Manning Publications Co.: Greenwich, CT, USA, 2003.

(4) *Communication from the European Commission to the European Council and the European Parliament: 20 20 by 2020: Europe's climate change opportunity*; European Commission: Brussels, Belgium, 2008.

(5) Cotton, F. A.; Wilkinson, G.; Murillio, C. A.; Bochmann, M. *Advanced Inorganic Chemistry*, 6th ed.; Wiley: Chichester, United Kingdom, 1999.

(6) Frisch, M. J. et al. Gaussian 03. Gaussian, Inc.: Wallingford, CT, 2004.

(7) (a) Johnson, A. L. (E. I. du Pont de Nemours). 1-(Alkylsubstituted phenyl)imidazoles useful in ACTH reverse assay. US Patent 3637731, 1972; (b) Arduengo, A. J., III; Dias, H. V. R.; Harlow, R. L.; Kline, M. Electronic stabilization of nucleophilic carbenes. *J. Am. Chem. Soc.* **1992**, *114*, 5530–5534; (c) Appelhans, L. N.; Zuccaccia, D.; Kovacevic, A.; Chianese, A. R.; Miecznikowski, J. R.; Macchioni, A.; Clot, E.; Eisenstein, O.; Crabtree, R. H. An anion-dependent switch in selectivity results from a change of C—H activation mechanism in the reaction of an imidazolium salt with $IrH_5(PPh_3)_2$. *J. Am. Chem. Soc.* **2005**, *127*, 16299–16311; (d) Arduengo, A. J., III; Gamper, S. F.; Calabrese, J. C.; Davidson, F. Low-coordinate carbene complexes of nickel(0) and platinum(0). *J. Am. Chem. Soc.* **1994**, *116*, 4391–4394.

(8) This is a note. The text will be moved the the references section. The title of the section will change to "Notes and References".

(9) Ref. 5, p. 1.

TOC Graphic

Some journals require a graphical entry for the Table of Contents. This should be laid out "print ready" so that the sizing of the text is correct.
Inside the `tocentry` environment, the font used is Helvetica 8 pt, as required by *Journal of the American Chemical Society*.
The surrounding frame is 9 cm by 3.5 cm, which is the maximum permitted for *Journal of the American Chemical Society* graphical table of content entries. The box will not resize if the content is too big: instead it will overflow the edge of the box.
This box and the associated title will always be printed on a separate page at the end of the document.
