# Supplementary figures and images for: Photoluminescence Enhancement in CdSe/CdS Quantum Dot Colloidal Films Induced by Gold Nanoparticles (AuNPs)
Source: ACS Omega. 2025 Sep 8;10(37):42472–9. doi: 10.1021/acsomega.5c03718 (PMC12461293; doi:10.1021/acsomega.5c03718)

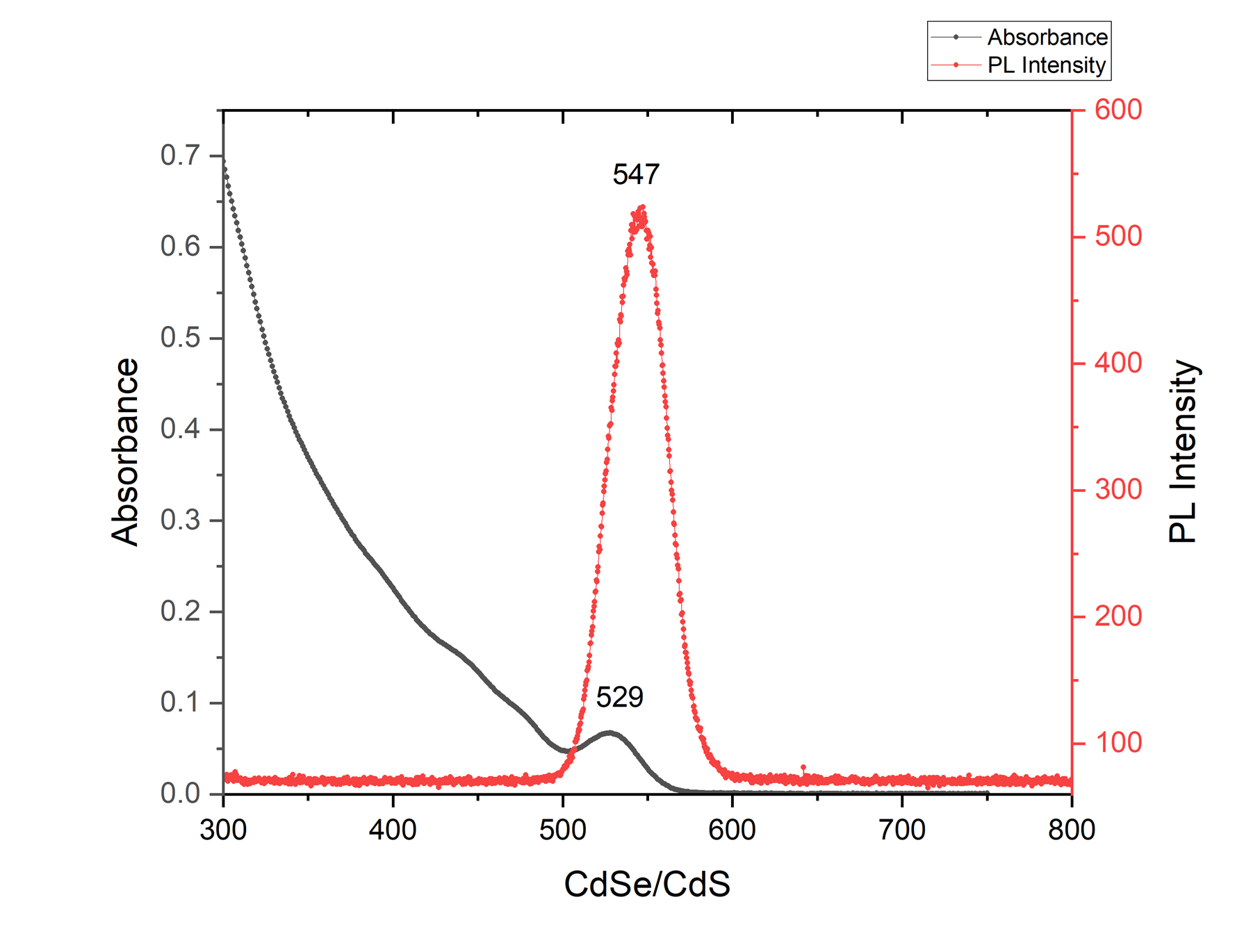

Supplement: Supplementary file 1 [file ao5c03718_si_001.zip › PL and Abs of QDs.png]

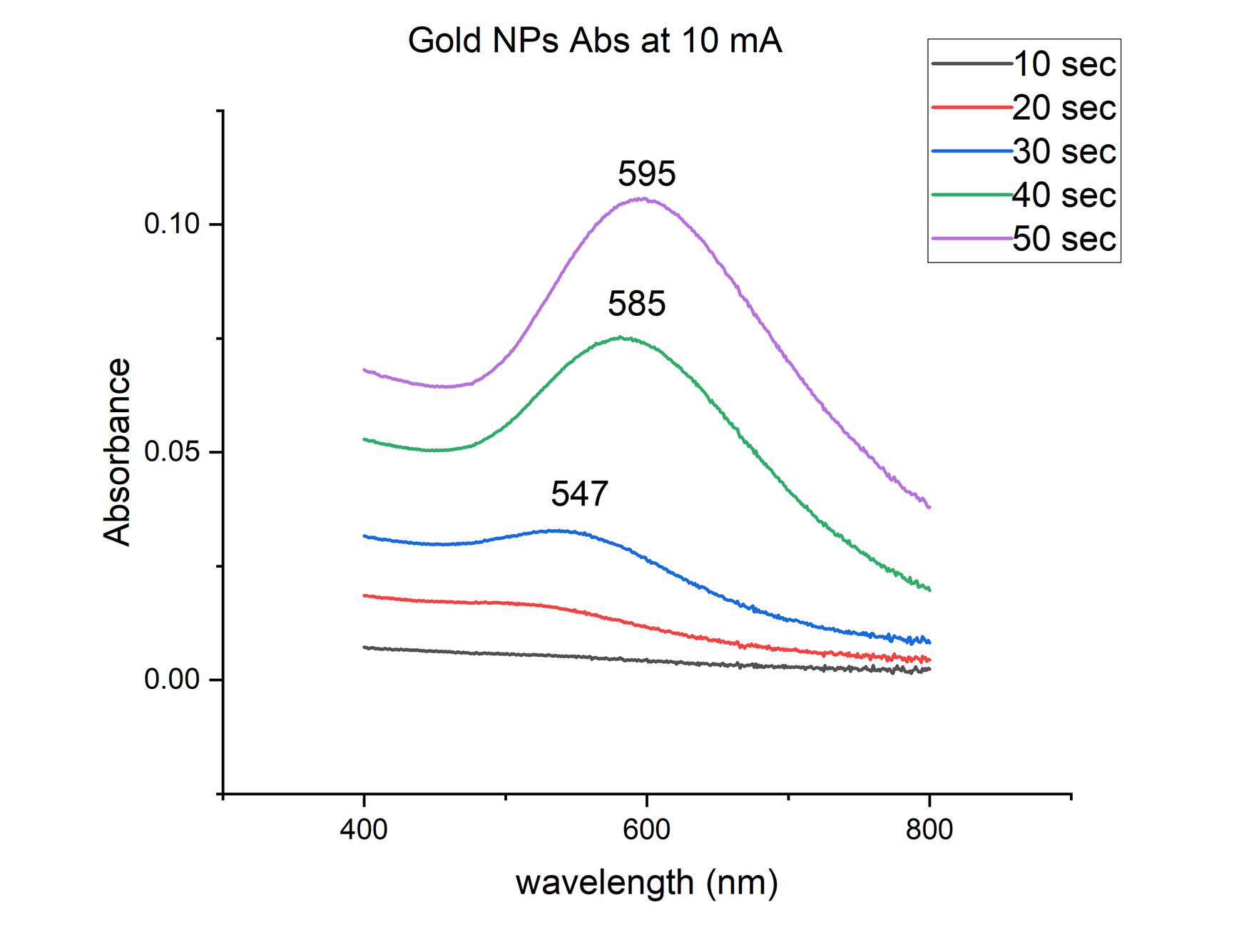

Supplement: Supplementary file 1 [file ao5c03718_si_001.zip › Abs specta of AuNPs for different time period.png]

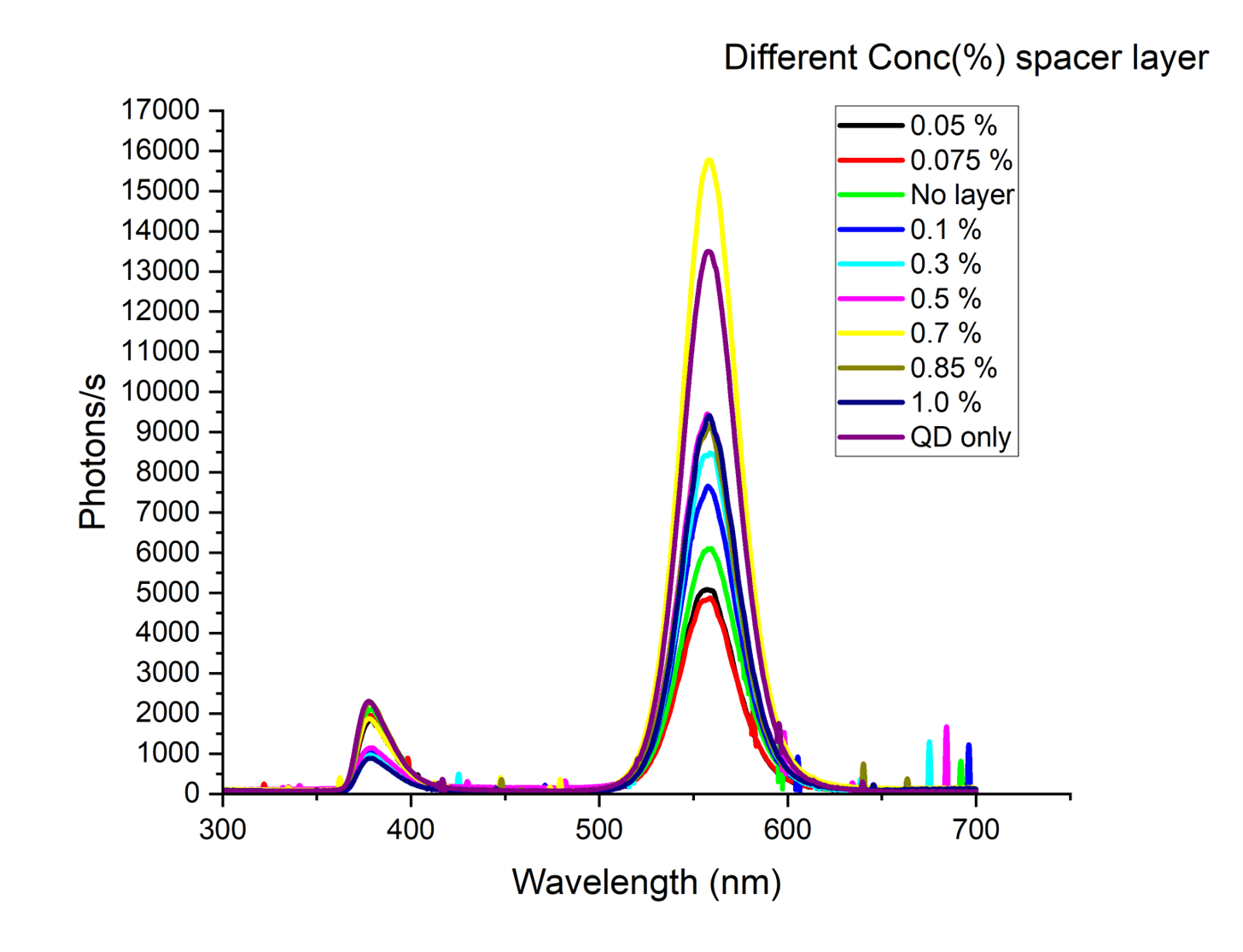

Supplement: Supplementary file 1 [file ao5c03718_si_001.zip › PL of coupling structure.png]

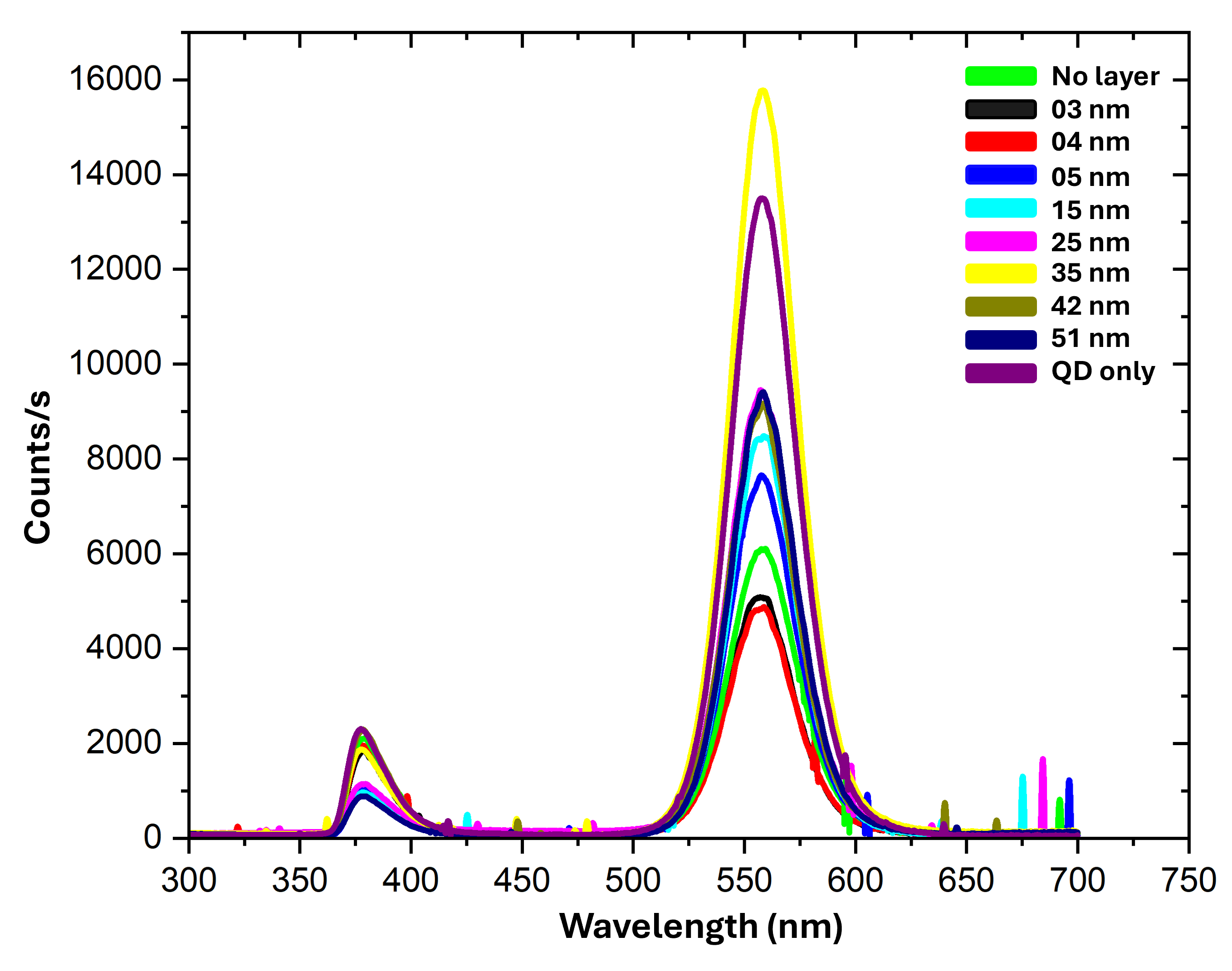

Supplement: Supplementary file 1 [file ao5c03718_si_001.zip › Pl raw data smoothed.jpg]

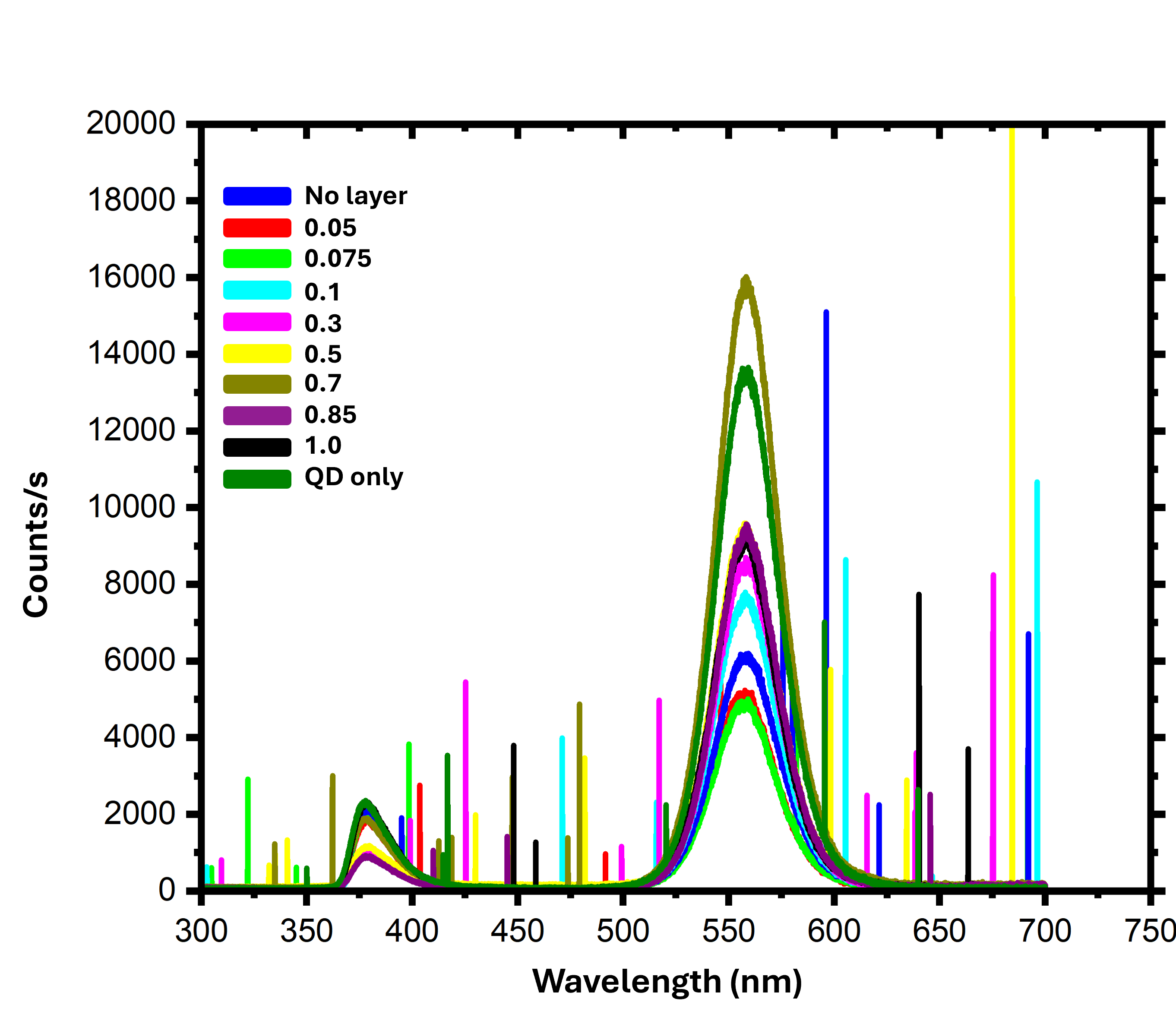

Supplement: Supplementary file 1 [file ao5c03718_si_001.zip › PL raw data.jpg]

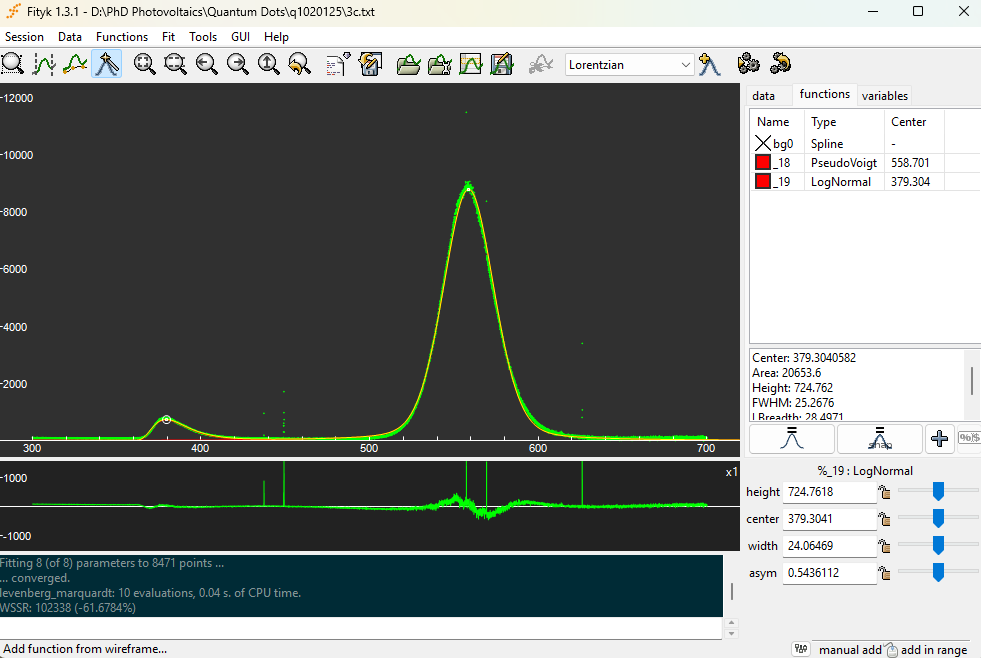

Supplement: Supplementary file 1 [file ao5c03718_si_001.zip › Fityk data fitting.jpg]
